# Antibacterial and antifungal activity of crude and freeze-dried bacteriocin-like inhibitory substance produced by *Pediococcus pentosaceus*

**DOI:** 10.1038/s41598-020-68922-2

**Published:** 2020-07-23

**Authors:** Pamela Oliveira de Souza de Azevedo, Carlos Miguel Nóbrega Mendonça, Ana Carolina Ramos Moreno, Antonio Vinicius Iank Bueno, Sonia Regina Yokomizo de Almeida, Liane Seibert, Attilio Converti, Ii-Sei Watanabe, Martin Gierus, Ricardo Pinheiro de Souza Oliveira

**Affiliations:** 1 Department of Biochemical and Pharmaceutical Technology, University of São Paulo, São Paulo, SP Brazil; 2 Department of Microbiology, Biomedical Sciences Institute, University of São Paulo, São Paulo, SP Brazil; 3 Department of Zootechnics, University of Maringá, Maringá, PR Brazil; 4 Department of Anatomy, Biomedical Sciences Institute, University of São Paulo, São Paulo, Brazil; 5 Department of Animal Science, Laboratory of Ecology and Natural Grassland, Federal University of Santa Maria, Santa Maria, RS Brazil; 6 Department of Civil, Chemical and Environmental Engineering, Pole of Chemical Engineering, University of Genoa, Via Opera 15, 16145 Genoa, Italy; 7 Department of Agrobiotechnology, Institute of Animal Nutrition, Livestock Products, and Nutrition Physiology (TTE), IFA-Tulln, University of Natural Resources and Life Sciences (BOKU), Vienna, Austria

**Keywords:** Biochemistry, Biotechnology, Microbiology

## Abstract

*Pediococcus pentosaceus* LBM 18 has shown potential as producer of an antibacterial and antifungal bacteriocin-like inhibitory substance (BLIS). BLIS inhibited the growth of spoilage bacteria belonging to *Lactobacillus*, *Enterococcus* and *Listeria* genera with higher activity than Nisaplin used as control. It gave rise to inhibition halos with diameters from 9.70 to 20.00 mm, with *Lactobacillus sakei* being the most sensitive strain (13.50–20.00 mm). It also effectively suppressed the growth of fungi isolated from corn grain silage for up to 25 days and impaired morphology of colonies by likely affecting fungal membranes. These results point out that *P. pentosaceus* BLIS may be used as a new promising alternative to conventional antibacterial and antifungal substances, with potential applications in agriculture and food industry as a natural bio-controlling agent. Moreover, cytotoxicity and cell death induction tests demonstrated cytotoxicity and toxicity of BLIS to human colon adenocarcinoma Caco-2cells but not to peripheral blood mononuclear cells, with suggests possible applications of BLIS also in medical-pharmaceutical applications.

## Introduction

Lactic acid bacteria (LAB) are a group of well-distributed microorganisms in nature^1–3^ having lactic acid as the major product of sugar fermentation^4,5^. They are Gram-positive, non-pathogenic, non-sporulating, facultative anaerobic, catalase negative and acid tolerant bacteria with a strictly fermentative metabolism^3,6^, which are often used as industrial starter cultures in food fermentation technology^7,8^. Their metabolic products are recognized as Generally Regarded as Safe (GRAS) by the Food and Drug Administration (FDA) and recommended by the Qualified Presumption of Safety (QPS) list of the European Food Safety Authority (EFSA)^9^. This means that they can be safely used to preserve foods^10,11^, hence offering important benefits to the food industry^12^.

From the technology point of view, the most important genera of LAB are *Aerococcus*, *Carnobacterium*, *Enterococcus*, *Lactobacillus*, *Lactococcus*, *Leuconostoc*, *Oenococcus*, *Pediococcus*, *Streptococcus*, *Tetragenococcus*, *Vagococcus* and *Weissella*^13,14^. In addition to the acidic conditions ensured in foods, these microorganims also produce several antimicrobial agents like hydrogen peroxide, ethanol, diacetyl, carbon dioxide and bacteriocins, which may be exploited to preserve foods^1,15^ and livestock feeds such as silage. Inadequade fermentation and poor feed-out management of silage were in fact reported to lead to its spoilage and flavor proliferation and transmission of pathogens^16–18^.

Bacteriocins are ribosomally-synthesized peptides exerting their antimicrobial activity against either strains of the same species or against species more distantly related to the bacteriocin producer^19–21^. Since these compounds are easily accepted by consumers because of their natural origin^15^, studies on their production and application have been increasing in the last years. BLIS is the term recommended for not yet completely characterized antimicrobial peptides or proteins with unusual amino acids, having different chemical structures^22^ and exerting bactericidal or bacteriostatic action against Gram-positive and Gram-negative bacteria, without affecting the producer^23–27^. Bacteriocins and BLIS have cationic and amphiphilic structures with variable physicochemical characteristics in terms of molecular mass, amino acid sequence and isoelectric point^24,28,29^. Normally they undergo post-translational modifications and are released to the outer environment or remain bound to cell membrane of the producing bacterium^24^.

Nisin, the most studied bacteriocin, is authorized by FDA for use in foods and regulated as a food additive in 48 countries to preserve products such as milk, dairy products, tomato and other canned vegetables, canned soups, mayonnaise and infant foods^30,31^. It is produced by several strains of *Lactococcus lactis* subsp. *lactis*^10,27,32,33^ and commercialized by DuPont Danisco under the trade name Nisaplin. Nisaplin has a broad spectrum of action against Gram-positive bacteria, including LAB^34^, but, generally, it is hardly effective against Gram-negative bacteria, molds and yeasts^30,31^.

Another bacteriocin with promising future as food industrial preservative^35,36^ is pediocin, which is produced by members of the genus *Pediococcus*. Several pediocins have been characterized^37^, which may be used as preservatives of foods for humans and farm animals, including vegetables, meat products, cheese and grass or corn silages^38^. They exhibit important technological properties such as thermostability and capacity to retain antimicrobial activity in a wide range of pH, especially against Gram-positive food spoilage and foodborne pathogenic bacteria^37,39^, but also against yeasts, molds, filamentous fungi and mushrooms^40^.

The aim of this study was to evaluate the efficiency of BLIS produced by *Pediococcus pentosaceus* LBM 18, either in its crude or freeze-dried form, as a promising new preservative against fungi in corn grain silage.

## Materials and methods

### Microbial cultures

The BLIS-producing strain *Pediococcus pentosaceus* LBM 18 was isolated from corn silage, while all media were acquired from Roth (Karlsruhe, Germany). It was cultivated in commercial de Man, Rogosa and Sharp (MRS) medium at 30 °C for 10 h in an incubator without stirring. *Lactobacillus sakei* ATCC 15521, *Enterococcus faecium* 2052, *Listeria innocua* NCTC 11288 and *Listeria seeligeri* NCTC 11289 were used as indicator strains. *L. sakei* and *E. faecium* were overnight cultivated at 37 °C in MRS medium, while *Listeria* strains in Brain Heart Infusion (BHI) medium under the same conditions. Fungi isolated from Austrian corn grain silage^41^ were cultivated in Potato Extract Glucose (PEG) medium at 30 °C in an incubator without stirring for at least three days, since fast-growing fungi can be detected after two days of cultivation^42^. Following manufacturer’s instructions, all the culture media were autoclaved (2,540 ELV, Tuttnauer, Hauppauge, NY, USA) at 121 °C for 12 min (MRS) or 15 min (BHI and PEG).

## Cultivations for BLIS production

BLIS was produced in static *P. pentosaceus* cultivations carried out at 30 °C for 10 h in 500-mL Erlenmeyer flasks containing 300 mL of MRS medium placed in an incubator. BLIS-containing medium was separated from *Pediococcus* biomass by centrifugation (4,470 × g at 4 °C for 20 min), and the supernatant pH adjusted to 6.0–6.5 by addition of 1.0 N NaOH for use in the analyses. Crude BLIS (CB), i.e. BLIS without any purification, was tested for its activity against the above indicator strains after several dilutions with sterile deionized water, namely 1:2, 1:5, 1:10, 1:50 and 1:100 (v/v). Freeze-dried crude BLIS (FB) was tested at three different concentrations, namely 1.0, 2.5 and 5.0% (w/v), after its dilution with sterile deionized water.

Nisaplin (DuPont Danisco, Copenhagen, Denmark), having nisin in its formulation as an active compound at 2.5% (w/w), was also diluted with sterile deionized water up to the same concentrations as FB (1.0, 2.5 and 5.0%, w/v) and used for comparison with BLIS antimicrobial power.

## Determination of BLIS antibacterial activity

The agar-well diffusion method was performed to evaluate the antimicrobial activity of CB, FB and Nisin against *L. sakei*. For this purpose, after overnight bacterial growth at 37 °C, 150 µL of each microbial suspension with 0.3 optical density at 600 nm (OD_600nm_), corresponding to 8 × 10^6^ CFU/mL for *L. sakei* and 3 × 10^6^ CFU/mL for *E. faecium*, *L. innocua* and *L. seeligeri*, were 1:100 (v/v) diluted with sterile deionized water, added to 15 mL of MRS soft agar-medium (0.75%, w/v) and poured into Petri dishes. After agar-medium solidification, 50 μL of BLIS or Nisin were added into each well, plates were incubated at 37 °C for 16–18 h, and the antagonistic activity was determined by measuring the diameter of the inhibition halos in millimeters^43^.

The antagonistic activity of CB without dilution or diluted several times [from 1:2 to 1:100 (v/v)] was also evaluated by counting colonies according to the pour plate methodology. All the assays were performed in triplicate.

## Determination of BLIS antifungal activity

Fungi isolated from Austrian corn grain silage^41^ were grown in PEG medium in an incubator at 30 °C for 3–5 days. After incubation, CB was applied directly on the different mycelia, and the seven fungi that proved to be sensitive to CB were isolated from the plates, named from F1 to F7 and grown separately. Sensitive fungi were evaluated by the pour plate technique, after addition of 1.0 mL of sample, consisting of 500 µL of fungus suspension and 500 µL of CB (or 500 µL of sterile deionized water in the control), to 10 mL of Yeast Extract Glucose (YEG) agar medium (Merck Millipore, Darmstadt, Germany), and incubation at 30 °C for 24 h, 7 days, 15 days and 25 days. After these periods, mycelial growth in both treated and control Petri dishes were visually examined.

## BLIS exposure to hydrolytic enzymes

The effect of various hydrolytic enzymes on BLIS activity was also tested. Aliquots of BLIS solution (1.4 mL) were incubated with 100 µL of 2.0 mg/mL trypsin, pepsin, papain (Interlab, São Paulo, SP, Brazil), proteinase K or α-amylase (Sigma-Aldrich, St. Louis, MO, USA) at 37 °C for 1 h. Samples were placed in water bath at 90 °C for 3 min for enzyme inactivation. Untreated BLIS served as a control.

## Protein quantification

Protein quantification of CB and solutions of BLIS (1:2, 1:5, 1:10, 1:50, 1:100, 1:250, 1:500, 1:1,000 and 1:5,000), diluted in sterile deionized water (v/v), was performed using the Bicinchoninic Acid Protein Assay Kit BCA1 (Sigma-Aldrich, St. Louis, MO, USA) according to the manufacturer. For comparison, commercial pediocin from *Pediococcus acidilactici* (P0098, Sigma-Aldrich, St. Louis, MO, USA) was also used. The unknown protein content of samples (*C*_P_) was assessed by means of absorbance readings at 560 nm (Abs) and a calibration curve (*C*_P_ = 0.0015 Abs + 0.431; R^2^ = 0.992) previously constructed using Kit reagents with known protein concentration.

## Physical characterization of BLIS

Fourier Transform Infrared Spectroscopy (FT-IR) (Frontier—PerkinElmer, Massachusetts, USA) was used for a partial physical characterization of BLIS, since it allows qualitatively identifying unknown functional chemical groups^43,44^ in any different kind of material. For this purpose, 1.0 mg of lyophilized BLIS was milled with 100 mg of KBr at a pressure of 7,500 kg cm^-2^ per 30 s, and the resulting translucent disks were analyzed by FT-IR ^45^ in the 4,000–400 cm^-1^ wavenumber range with resolution of 4 cm^-1^.

CB dissolved in deuterated water was also analyzed by ^1^H Nuclear Magnetic Resonance (NMR) with a Bruker AIII 500 spectrometer (Bruker, Rheinstetten, Germany) at 500 MHz, to find out different amino acids that form the molecular structure of BLIS^46^. The ^1^H chemical shifts were expressed in ppm with respect to the standard solvent displacement.

## Scanning electron microscopy

To examine *P. pentosaceus* cells, Scanning Electron Microscopy (SEM) was used as previously described^47^. For this purpose, samples were prepared as follows. After cultivation, the culture medium was removed by centrifugation at 5,000 rpm and 4 ºC for 20 min. In order to remove contaminants from the culture medium and free biomass from the viscous matrix formed during fermentation, the pellet obtained was redispersed in 100 mL of deionized water and subjected to several cycles of centrifugation under the same conditions described above and washing with an equal water volume, until a milky color liquid was obtained. The viscous matrix was then redispersed in 50 mL of deionized water and cryogenically frozen by immersion and cooling to a temperature of − 196 ºC using liquid nitrogen. The sample water was subsequently removed by lyophilization, and the resulting white solid with flocculated consistency was preserved in a desiccator at room temperature for further analysis. The samples were mounted on metal stubs with a conductive paste and coated with gold or gold–palladium in an argon-ion atmosphere, and examined in a Scanning Electron Microscope, model Neoscope JCM-5000 (JEOL, Peabody, MA, USA).

## Cytotoxicity of BLIS to human cells

To assess the cytotoxicity of BLIS to human cells, peripheral blood mononuclear cells (PBMC) and human colon adenocarcinoma cells (Caco-2) were used^48^. In 96-well plates, 2.5 × 10^5^ cells per well were plated in 100 µL of culture medium [RPMI (Gibco, Grand Island, NY, USA) for PBMC cells or DMEN (Gibco) for Caco-2 cells] supplemented with 10% fetal bovine serum. Plates were incubated in a 5% CO_2_ oven at 37 °C for 24 h to allow cell adhesion. After 24 h of incubation, crude BLIS without any dilution was added to the wells, and plates were incubated again for 48 h. The control was prepared in the same way but without adding the BLIS. Each test was performed in triplicate. After incubation, the content of each well was removed, and 100 µL of 20 µg/ml 3-(4,5-dimethylthiazol-2yl)-2,5-diphenyl tetrazoline bromide (MTT) were added. Plates were incubated for 3 h under the same conditions mentioned above. The MTT solution was then removed from the wells, and 200 µL of dimethyl sulfoxide (DMSO) were added. After smooth homogenization of plate content to dissolve formazan crystals, the absorbance was read at a wavelength of 570 nm.

## Cell death induction assay

After cultivation, removal of the culture medium and treatment with BLIS as described in the previous section, cells were stained with 100 µL of 50 µg/mL acridine orange solution and 50 µg/mL propidium iodide prepared in phosphate saline buffer (PBS). After 5 min of incubation, acquisition of images was done using a fluorescence microscope (Axiovert 200, Zeiss, Oberkochen, Germany). According to this methodology, viable cells stained green, due to the presence of acridine orange, while dead cells stained red because of propidium iodide.

## Statistical analysis

The results, expressed as mean values ± standard deviations, were submitted to one-way analysis of variance (ANOVA) by the Statistica Software 12 (TIBCO Software Inc., Palo Alto, CA, USA), compared using the Tukey’s post-hoc test and considered significantly different when *p* < 0.05.

## Results and discussion

### Antibacterial activity

The ability of *Pediococcus pentosaceus* LBM 18 to produce bacteriocin-like inhibitory substance (BLIS) and its effectiveness as an antimicrobial agent were first evaluated by the agar well diffusion assay. As shown in Table 1, all the indicator strains were sensitive to BLIS, exhibiting large diameters of the inhibition halos (9.70 to 20.00 mm), with *Lactobacillus sakei* being the most sensitive one (13.50 to 20.00 mm), regardless of BLIS dilution. Even though BLIS was used in its crude form (CB), it showed higher antimicrobial activity (larger inhibition halos) against all indicator strains compared to 1% (w/v) nisin-based Nisaplin solution.

**Table 1:** Inhibition of growth of bioindicator strains after treatment with 1% (w/v) Nisaplin or Crude BLIS (CB) with or without dilution, expressed as diameter of inhibition halo (mm) or counts of colony forming units (log CFU/mL).

Inhibition	Antimicrobial and dilution (v/v)	Bioindicator strains			
		Ls15521	En2052	Li11288	Lse11289
Halo diameter (mm)	CB	20.00 ± 0.20	14.50 ± 0.10	15.00 ± 0.10	14.00 ± 0.10
	1:2 CB	19.00 ± 0.40	15.80 ± 0.30	15.00 ± 0.20	15.50 ± 0.20
	1:5 CB	18.00 ± 0.30	16.70 ± 0.20	15.70 ± 0.30	16.00 ± 0.10
	1:10 CB	15.00 ± 0.20	17.00 ± 0.20	16.50 ± 0.10	17.00 ± 0.20
	1:50 CB	14.00 ± 0.20	14.50 ± 0.10	11.00 ± 0.10	14.50 ± 0.10
	1:100 CB	13.50 ± 0.30	12.00 ± 0.10	10.00 ± 0.10	13.50 ± 0.10
	Nisaplin	13.30 ± 0.40	12.50 ± 0.20	9.70 ± 0.10	12.50 ± 0.20
Counts (logCFU/mL)	C	7.54 ± 0.08	7.02 ± 0.04	8.25 ± 0.06	8.21 ± 0.06
	CB	˂ 4	˂ 4	˂ 4	7.81 ± 0.10
	1:2 CB	˂ 4	˂ 4	˂ 4	˂ 4
	1:5 CB	˂ 4	˂ 4	7.96 ± 0.10	˂ 4
	1:10 CB	˂ 4	˂ 4	8.22 ± 0.00	7.47 ± 0.00
	1:50 CB	˂ 4	˂ 4	8.11 ± 0.29	8.27 ± 0.06
	1:100 CB	˂ 4	˂ 4	8.11 ± 0.31	8.27 ± 0.06
	Nisaplin	˂ 4	˂ 4	˂ 4	˂ 4

C: control (water), Ls15521: *Lactobacillus sakei* ATCC 15521, En2052: *Enterococcus faecium* 2052, Li11288: *Listeria innocua* NCTC 11288, Ls11289: *Listeria seeligeri* NCTC 11289. Values are the means of triplicates ± standard deviations.

BLIS was shown to be more effective when diluted in water, especially at 1:5 and 1:10 (v/v) dilutions (Table 1), likely because of better diffusion through the bacterial cell membrane. At the highest dilutions (1:50 and 1:100), BLIS still exerted a weak antibacterial effect against all strains, with significantly (*p* < 0.05) narrower inhibition halos (10.00 to 14.50 mm).

BLIS antibacterial effect was also checked by counting colonies according to the pour plate method, whose results are listed in Table 1. Both CB and BLIS at any dilutions were as effective as Nisaplin in suppressing *L. sakei* and *E. faecium* growth (logCFU/mL < 4), irrespective of dilution. *Listeria* strains proved to be less sensitive, in that *L. innocua* growth was completely suppressed (logCFU/mL < 4) only by CB and 1:2 (v/v) water-diluted BLIS and *L. seeligeri* by 1:2 and 1:5 (v/v) water-diluted BLIS. The strain-dependent efficiency of CB or water-diluted BLIS may be explained by interaction between BLIS and plasma membrane and consequent formation of pores, which is a common occurrence among most LAB bacteriocins. BLIS may have also interacted with receptors or docking molecules, in a specific way for a specific strain depending on its membrane lipid composition and membrane potential threshold^49^, abrogating essential cell pathways which account for BLIS potency^50^. At the other BLIS dilutions (1:10, 1:50 and 1:100, v/v), *L. innocua* showed growth (logCFU/mL = 8.22 ± 0.00, 8.11 ± 0.29, 8.11 ± 0.31, respectively) similar to that in the control (logCFU/mL = 8.25 ± 0.06). Also, *L. seeligeri*, when exposed to 1:50 and 1:100 (v/v) diluted BLIS, practically achieved the same counts (logCFU/mL 8.27 ± 0.06) as in the control (logCFU/mL = 8.21 ± 0.06). These results confirm the known broad spectrum of action of *P. pentosaceus* bacteriocins, especially against the members of *Listeria* genus. To provide only a few examples, the antimicrobial activity of *P. pentosaceus* BLIS was proven against different strain of *L. monocytogenes*. In particular, Yin et al.^51^ isolated from pork meat two *P. pentosaceus* bacteriocins, named as pentocins L and S, which exhibited antimicrobial activity against *L. monocytogenes* RII, LM and CCRC 14845. The antimicrobial activity of bacteriocins produced by *P. pentosaceus* was also reported against *Staphylococcus aureus* 196E^52^, spores of several *Clostridium botulinum* strains^53^, *C. botulinum* ATCC 11259^51^ and LAB such as *Lactobacillus plantarum* LB 75 and LB 592, *L. sakei* LB 706 and LB 592^54^, which suggests the use of this microorganism to produce novel antimicrobials.

The efficiency of BLIS as an antibacterial was also tested after freeze-drying (Table 2) to check whether its antimicrobial activity would be impaired by this operation. Regardless of BLIS concentration (1.0, 2.5 or 5.0%, w/v), its ability to reduce *L. sakei* counts (logCFU/mL) was in the range 0.5–0.6, which suggests the existence of a threshold amount for bacteriocin to exert a bacteriostatic or bactericidal effect against closely related species like *L. sakei*, while Nisaplin was much more effective, being able to completely suppress it (logCFU/mL < 4) regardless of its concentration (1.0, 2.5 and 5.0%, w/v) or dilution.

**Table 2:** Antibacterial activity against *Lactobacillus sakei* ATCC 15521 of freeze-dried BLIS or Nisaplin at different concentrations with or without dilution, expressed as counts of colony forming units (log CFU/mL).

Dilution (v/v)	Freeze-dried BLIS			Nisaplin		
	1.0% (w/v)	2.5% (w/v)	5.0% (w/v)	1.0% (w/v)	2.5% (w/v)	5.0% (w/v)
C	8.16 ± 0.10	8.16 ± 0.10	8.16 ± 0.10	8.16 ± 0.10	8.16 ± 0.08	8.16 ± 0.10
WD	8.15 ± 0.07	7.96 ± 0.42	8.06 ± 0.18	˂ 4	˂ 4	˂ 4
1:2	7.54 ± 0.08	7.81 ± 0.04	8.10 ± 0.04	˂ 4	˂ 4	˂ 4
1:5	7.54 ± 0.08	7.62 ± 0.21	7.52 ± 0.00	˂ 4	˂ 4	˂ 4
1:10	7.74 ± 0.12	7.62 ± 0.21	7.70 ± 0.08	˂ 4	˂ 4	˂ 4
1:50	7.58 ± 0.15	7.74 ± 0.05	7.71 ± 0.33	˂ 4	˂ 4	˂ 4
1:100	7.65 ± 0.15	7.60 ± 0.21	7.54 ± 0.08	˂ 4	˂ 4	˂ 4

C: control (water), WD: without dilution. Values are the means of triplicates ± standard deviations.

In summary, the results of this part of the study clearly show that *P. pentosaceus* LBM 18 BLIS exerted higher antimicrobial effect when directly used after its production rather than after freeze-drying. Moreover, BLIS proved its antimicrobial action even without any purification, showing potential as a novel biopreservative alternative to nisin-based preparations.

## Antifungal activity of BLIS

LAB were shown to produce, during growth, antifungals, inhibitors of mycotoxin synthesis and mycotoxin detoxifying agents, which are in great demand by the agricultural and food sectors^9^. It has been proven their ability to produce fatty acids^55^, cyclic dipeptides^56^, proteinaceous compounds^57^, organic acids^58–60^, bacteriocins and BLIS^61^ with potent antifungal effect^62^, whose action seems to occur, in some cases, through damage of fungal membrane^63^, but it is still unknown in other cases.

In general, BLIS production by *P. pentosaceus* begins during the exponential growth phase^64^, reaches a maximum in the early stationary phase, and then quickly decreases; in contrast, Dalie et al.^65^ observed that the production of antifungal metabolites by the strain *P. pentosaceus* L006 was not growth dependent and achieved a maximum at the end of the stationary growth phase.

**Figure 1:**
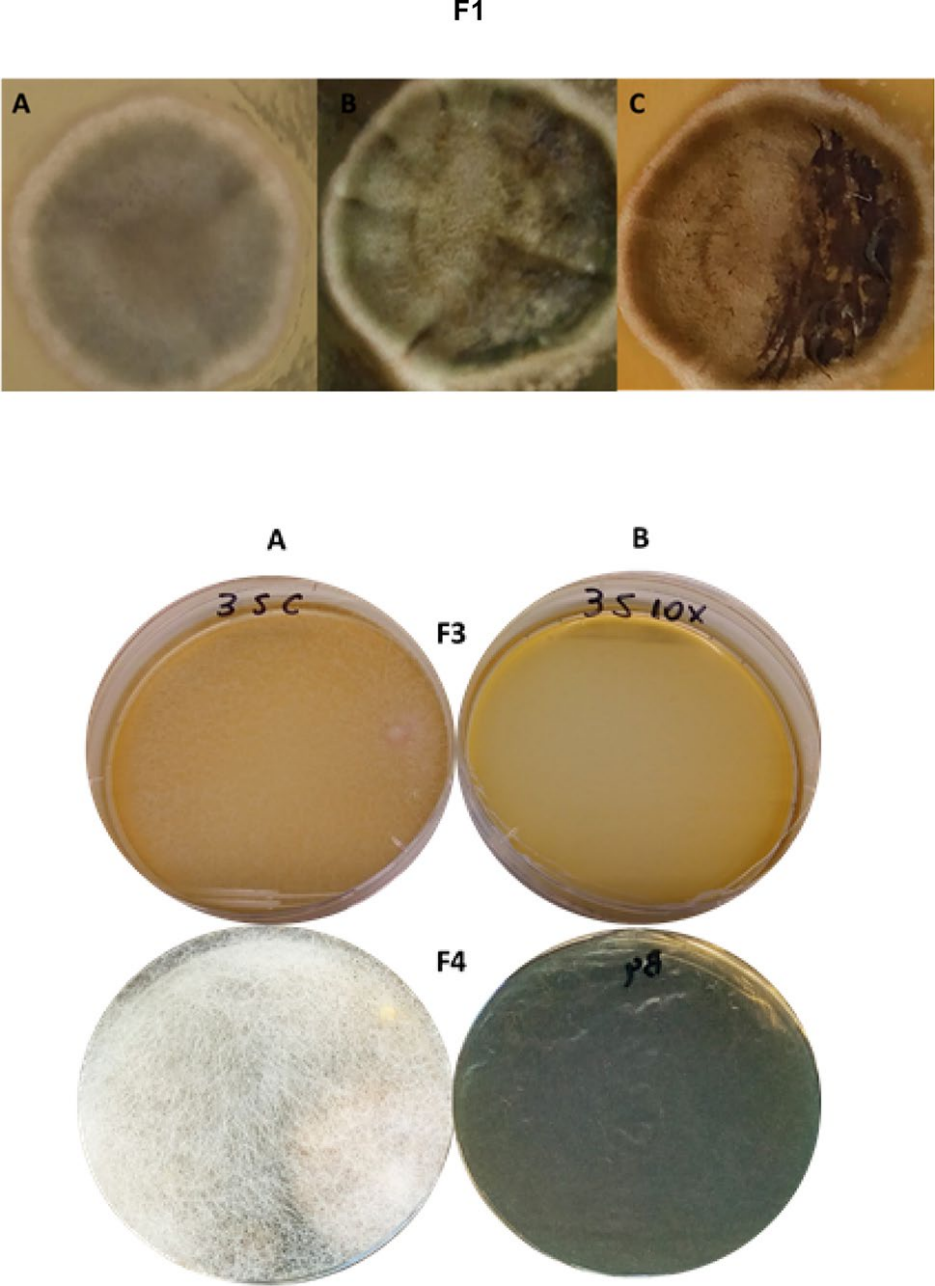
Antagonistic effect of crude BLIS produced by *Pediococcus pentosaceus* LBM 18 after 10 h of cultivation at 30 °C against the F1, F3 and F4 fungal isolates from Austrian corn grain silage. F1: (**a**) Before treatment; (**b**) 1 day after treatment; (**c**) 2 days after treatment. F3 and F4 after 3 months of treatment: (**a**) Control (water); (**b**) 1:10 (v/v) water-diluted BLIS.

*Aspergillus* spp. are spoilage microorganisms that are able to release mycotoxins in cereal grains^66^. To check the potential antifungal activity of *P. pentosaceus* LBM 18 BLIS, CB was applied directly to one of the fungal isolates (F1) from Austrian corn grain silage, which was apparently an *Aspergillus* (Fig. 1). Fungus damage was evident after 2 days of contact with CB from the prevention of mycelium expansion from the place of its application. This observation agrees with the antifungal effect observed against *Aspergillus niger* and *Aspergillus flavus* after exposure to cell-free culture of *P. pentosaceus* isolated from barley sourdough^67^.

Fungal spoilage and mycotoxin contamination are the greatest risks during storage of feed like silage. Silage is a traditional way to preserve farm animal feed for extended periods of time when feed is limited or unavailable^68,69^. It consists in green forage preservation by spontaneous lactic fermentation under anaerobic conditions^70,71^, whose primary purpose is to maximize nutritive value with minimum loss^71^. However, forage used for making silage is naturally in contact with yeasts and filamentous fungi present in the harvested biomass, or contamination may occur during harvesting, transport and storage. Growth of yeasts occurs under aerobic conditions, leading to loss of nutrients and dry matter, favoring the formation of butyric acid, reducing palatability and, consequently, shattering silage consumption^72^.

Most of studies published on this issue demonstrated a certain incidence of fungi in silage, which results in alteration or even deterioration of its nutritional value, being fungal distribution quite similar among different countries. However, the main fungi isolated from silage belong to *Aspergillus*, *Penicillium* and *Fusarium* genera, with high incidence of potentially toxicogenic species such as *A. flavus*, *Aspergillus parasiticus*, *Aspergillus fumigatus*, *Penicillium roqueforti*, *Fusarium verticilloides* and *Fusarium graminearum*^71^.

**Figure 2:**
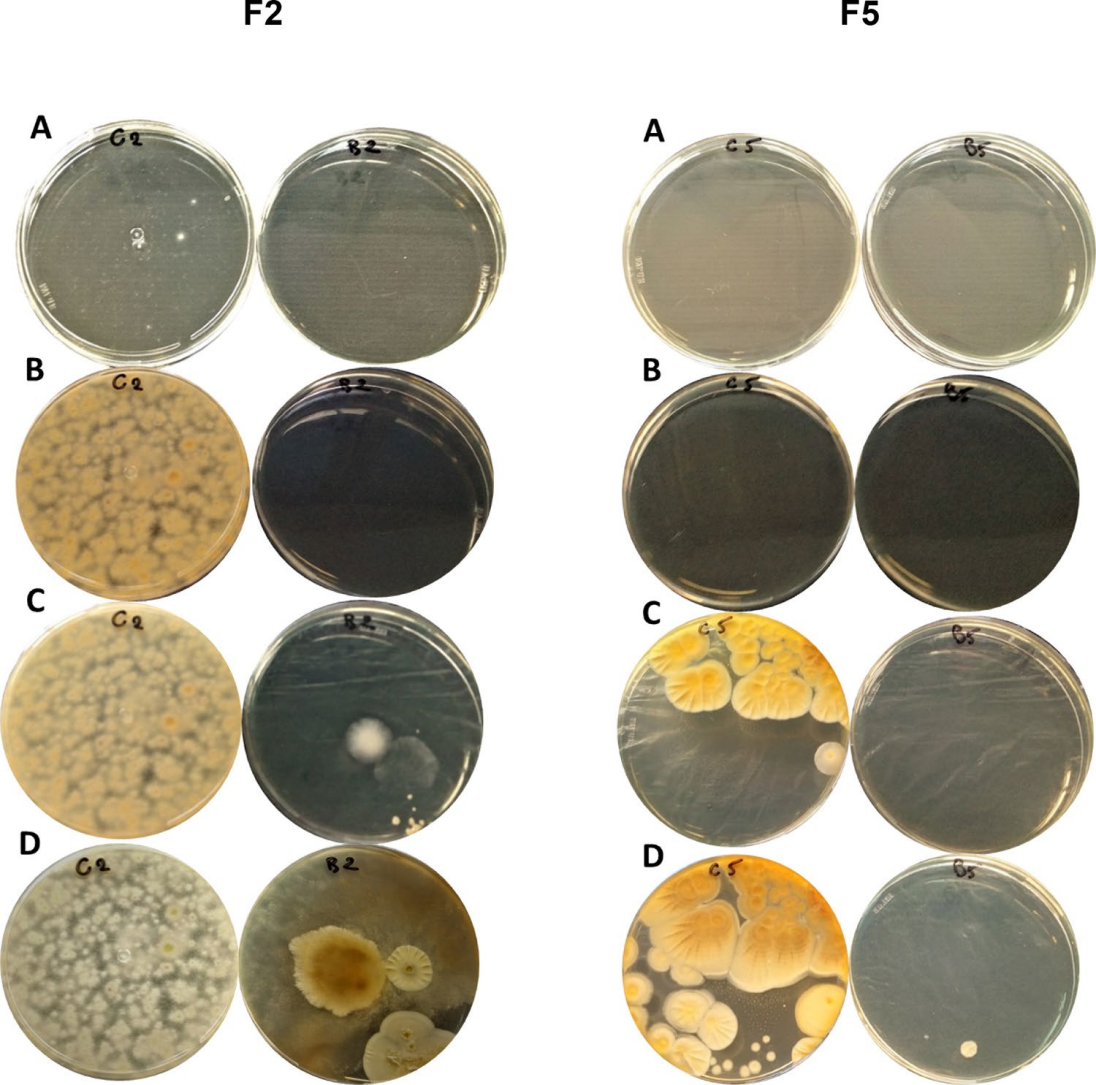
Antagonistic effect of BLIS produced by *Pediococcus pentosaceus* LBM 18 after 10 h of cultivation at 30 °C against the F2 and F5 fungal isolates from Austrian corn grain silage after (**a**) 24 h, (**b**) 7 days, (**c**) 15 days and (**d**) 25 days of treatment. (Left) Control (water); (Right) 1:5 (v/v) water-diluted BLIS.

**Figure 3:**
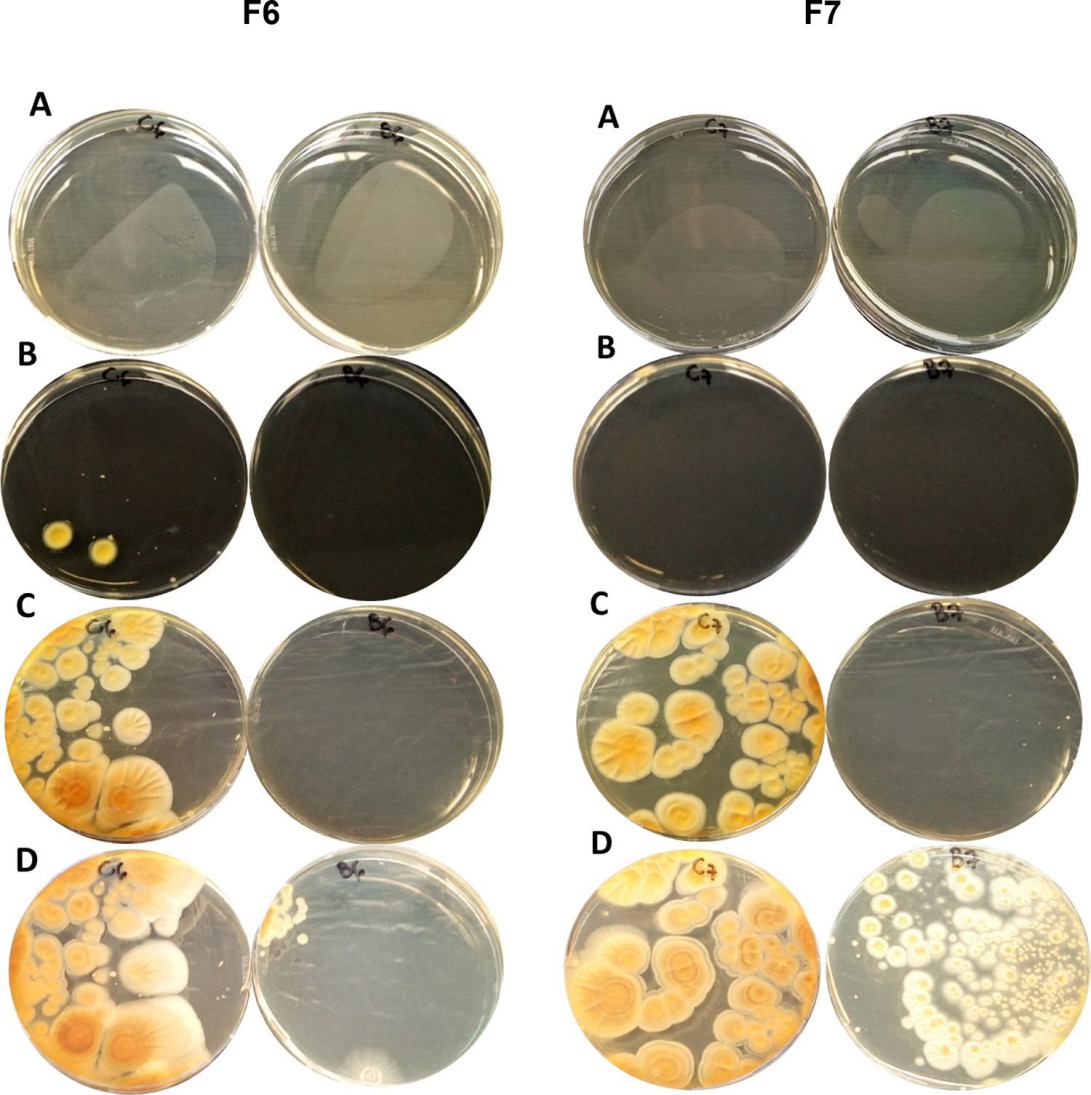
Antagonistic effect of BLIS produced by *Pediococcus pentosaceus* LBM 18 after 10 h of cultivation at 30 °C against the F6 and F7 fungal isolates from Austrian corn grain silage after (**a**) 24 h, (**b**) 7 days, (**c**) 15 days and (**d**) 25 days of treatment. (Left) Control (water); (Right) 1:5 (v/v) water-diluted BLIS.

In the present study, seven CB-sensitive fungi were isolated from Austrian corn grain silage^41^. Since CB was more effective as antibacterial agent after 1:5 and 1:10 (v/v) dilution with sterile deionized water (Table 1), both dilutions were tested against fungi. The 1:10 (v/v) water-diluted BLIS was very effective to inhibit the growth of two fungal isolates, named as F3 and F4, after 3-months of treatment (Fig. 1), while the 1:5 (v/v) one was effective against four other isolates, named as F2, F5 (Fig. 2), F6 and F7 (Fig. 3) until 7 and 15 days. After 25 days of 1:5 (v/v) water-diluted BLIS treatment, the F2, F5 (Fig. 2), F6 and F7 (Fig. 3) isolates were able to grow, but their growth was visibly impaired compared to the control (water). In addition, fungi that grew after 25 days of treatment had completely different morphology compared to the control, indicating a severe damage caused by the contact with BLIS. Such a damage may be ascribed to some effect of BLIS on fungal membrane and the observed variations to a sensitivity dependence on the species and/or strain, which may be related to different capacities to change the cell metabolism in response to stress conditions^63,67,72^.

These results agree with literature reports, in which *Pediococcus* genus has proven to be promising in the production of antifungal molecules^57,73^. For instance, *P. pentosaceus* L006 isolated from maize leaves was able to control the growth of mycotoxigenic molds such as a fumonisin-producing fungus^65^, while *Pediococcus acidilactici* LAB 5 isolated from meat was able to suppress the growth of food and feedborne molds and plant-pathogenic fungi^73^.

## Sensitivity of BLIS to hydrolytic enzymes

To assess the antibacterial activity of CB either before or after treatment with proteolytic enzymes, *L. sakei* was used as an indicator strain after 1:100 (v/v) dilution of a suspension with 0.3 OD_600nm_ in sterile deionized water. Non-treated CB exhibited an inhibition halo as large as 19.00 mm, while the treated one completely lost (100.0%) his activity (results not shown) after treatment with all proteolytic enzymes. Whereas the treatment with trypsin, pepsin and papain confirms the proteinaceous nature of BLIS, that with α-amylase suggests a complex structure^74^ with a saccharide moiety playing an important role in biological activity^75^. In addition, since there are only few examples of circular bacteriocins or BLIS resistant to proteolysis^76–78^, these results taken together indicate that BLIS produced by *Pediococcus pentosaceus* LBM 18 may be a linear proteinaceous glycoactive compound.

## Protein quantification

To obtain additional information on BLIS structure, CB protein content without any enzymatic treatment was determined after increasing dilutions (up to 1: 500 v/v) in sterile deionized water. As shown in Table 3, the 1:50 (v/v) and 1:100 (v/v) dilution allowed reducing CB protein content from 3.00 ± 0.74 g/mL to values (0.06 ± 0.01 g/mL and 0.04 ± 0.01 g/mL, respectively) close to that of commercial pediocins (0.05 g/mL), which are low molecular weight (2.7–4.0 kDa) peptides. Therefore, it can be inferred that the BLIS produced in this study may be a larger protein than commercial pediocins. Indeed, there are reports in the literature of bacteriocins with very large molecular size (> 10 kDa), which should then be classified as proteins rather than peptides^79–83^.

**Table 3:** Concentration (g/mL) of total proteins released from Crude BLIS (CB), diluted or without dilution, with or without enzymatic treatment, compared with commercial pediocin.

Samples	Protein concentration (g/mL)
CB	3.00 ± 0.74
1:2 (v/v) CB	2.21 ± 0.34
1:5 (v/v) CB	1.87 ± 0.71
1:10 (v/v) CB	1.45 ± 0.14
1:50 (v/v) CB	0.06 ± 0.01
1:100 (v/v) CB	0.04 ± 0.01
1:250 (v/v) CB	0.03 ± 0.00
1:500 (v/v) CB	0.03 ± 0.00
1:1,000 (v/v) CB	0.03 ± 0.00
1:5,000 (v/v) CB	0.02 ± 0.01
CB + trypsin	2.90 ± 0.81
CB + pepsin	2.91 ± 0.46
CB + papain	2.66 ± 0.71
CB + proteinase K	2.56 ± 0.51
CB + α-amylase	2.90 ± 0.96
Pediocin	0.05 ± 0.00

Values are the means of duplicates ± standard deviations.

## BLIS physical characterization

BLIS was partially characterized by Fourier Transform Infrared Spectroscopy (FT-IR) and ^1^H Nuclear Magnetic Resonance (^1^H NMR).

**Figure 4:**
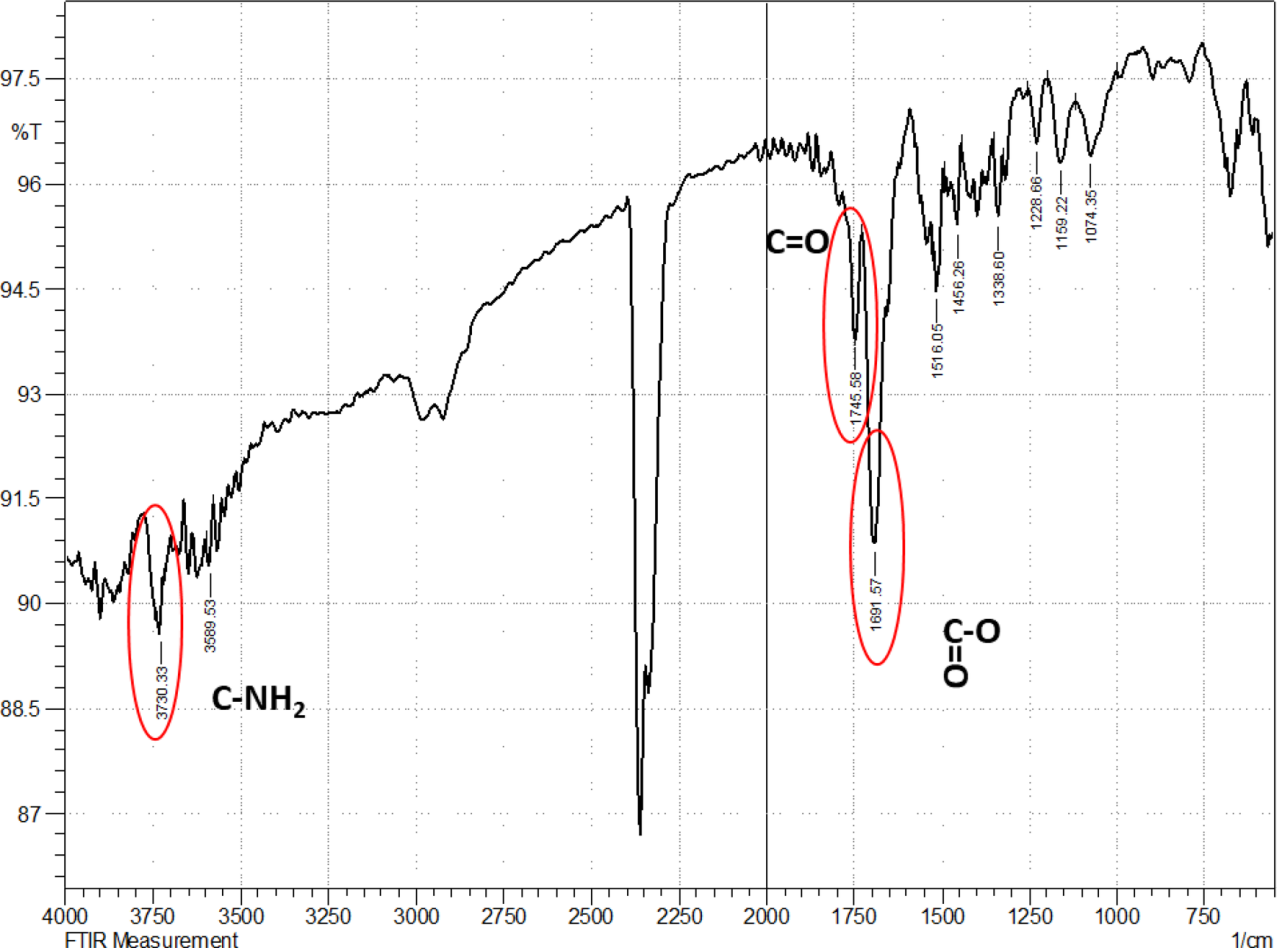
Fourier Transform Infrared Spectroscopy (FT-IR) spectrum of BLIS produced by *Pediococcus pentosaceus* LBM 18 after 10 h of cultivation.

FT-IR is a useful technique that allows identifying functional groups and then elucidating the chemical structure of unknown molecules. The FT-IR spectrum of BLIS produced by *P. pentosaceus* LBM 18 after 10 h of cultivation (Fig. 4) points out the presence of bands located at 1691 and 1745 cm^-1^ corresponding to carbonyls of carboxylic acids and amides, respectively, while those in the region of 1,700–1,550 cm^-1^ are characteristic of polysaccharides and are usually attributed to C=O stretching^84^. The absorption band at 3,730 cm^-1^ can be assigned to C-NH_2_ stretching^85^. Possibly, the stretching vibration at 1745 cm^-1^ may be attributed to the carbonyl bonded to the C-NH_2_ group, which would correspond to the presence of an amide in the biomolecule under study.

**Figure 5:**
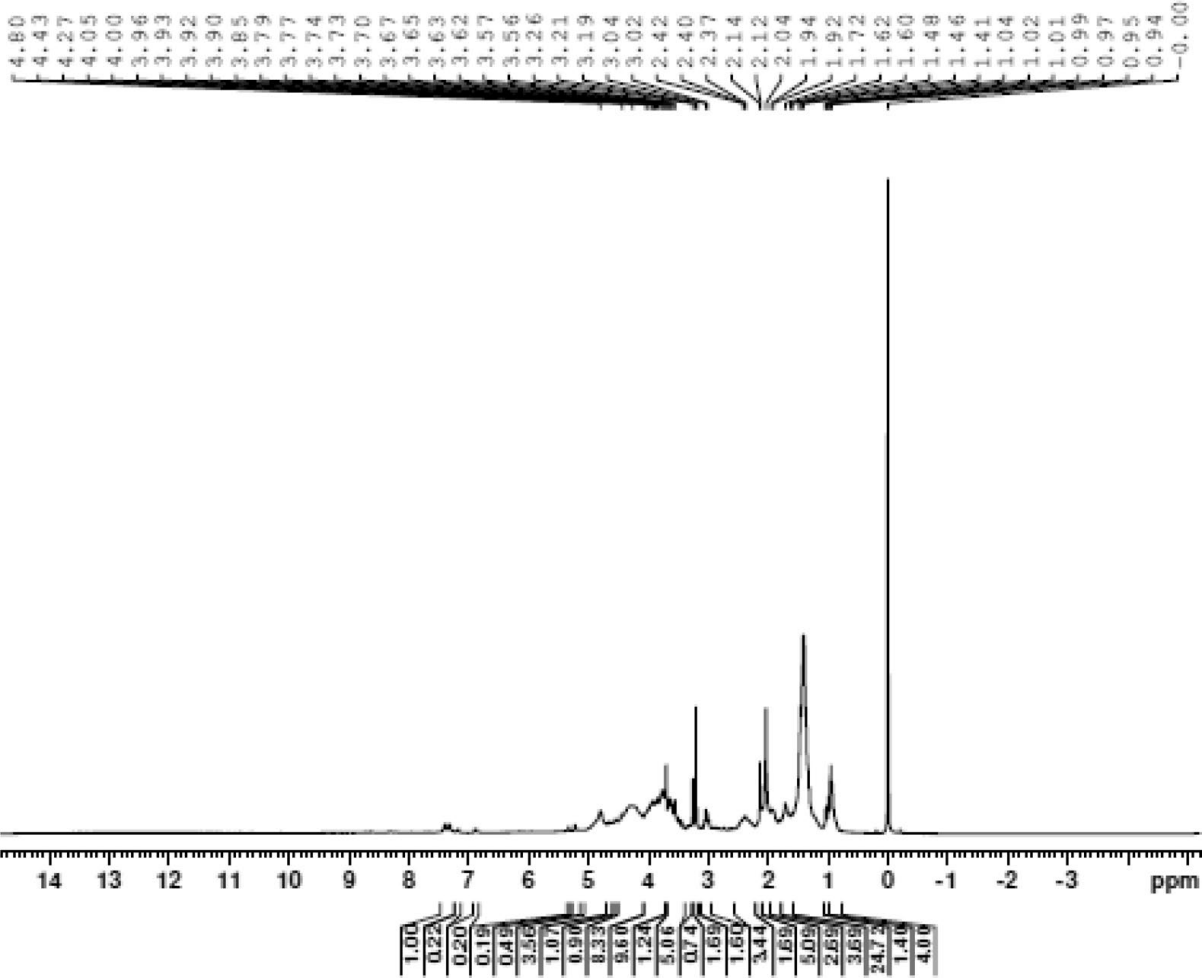
^1^H Nuclear Magnetic Resonance spectrum of BLIS produced by *Pediococcus pentosaceus* LBM 18 after 10 h of cultivation.

Chemical shifts of carbon and hydrogen atoms in NMR spectra give significant information about structures^85,86^. Some peaks (signals) in the ^1^H NMR spectrum (0.9, 1.3–1.5 and 2.0 ppm) correspond to the signals of protons of the aliphatic chains R-CH_3_, R-CH_2_-R and R-CH-R, respectively, while peaks between 3.3–4.0 ppm to CH_2_-O and H-C–OH groups (Fig. 5). Peaks between 7.0–7.5 ppm and 5.0–9.0 ppm correspond to the aromatic ring and amides, respectively^87^.

**Figure 6:**
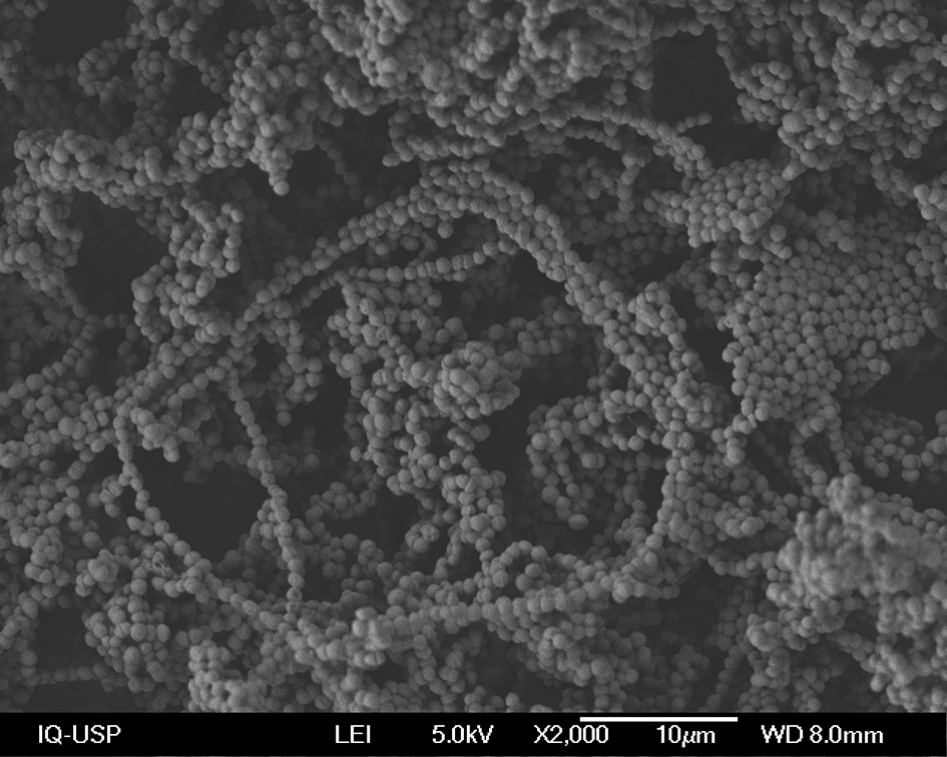
Micrograph of *Pediococcus pentosaceus* LBM18 cells obtained by Scanning Electron Microscopy. Magnification: × 2000.

While the amidic groups identified by FT-IR may be easily ascribed mainly to BLIS, the nature of polysaccharides required additional information, for which we resorted to Scanning Electronic Microscopy. One can see in Fig. 6 that *P. pentosaceus* LBM cells tended to form aggregates, which suggests that at least a significant portion of them may be constituted by exopolysaccharides typically involved in the formation of biofilms by species of the same genus^88^.

## Cytotoxicity of BLIS in human cells

**Figure 7:**
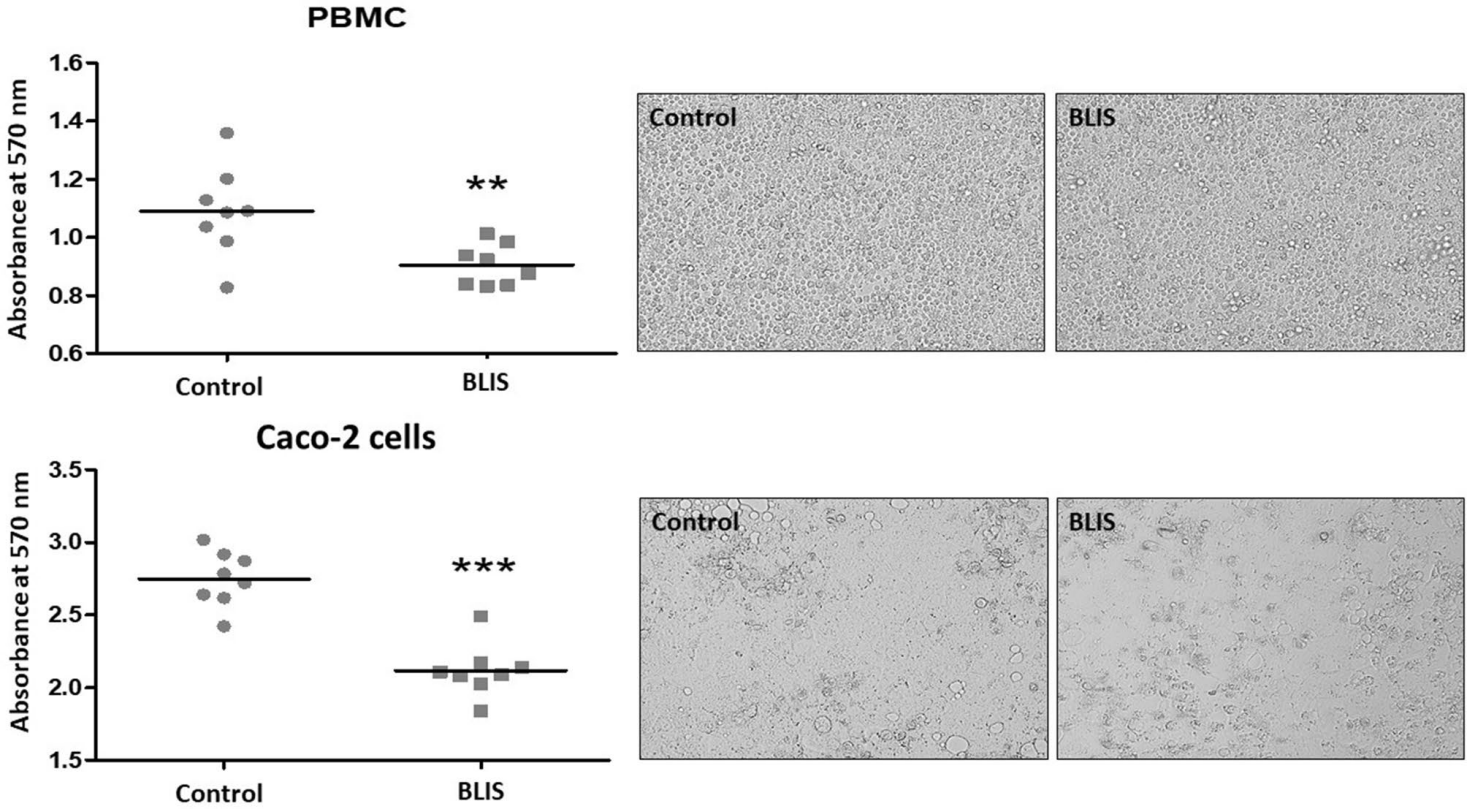
Cytotoxicity profile of (**a**) peripheral blood mononuclear cells (PBMCs) and (B) human colon adenocarcinoma Caco-2 cells after 48 h of treatment with the BLIS produced by *P. pentosaceus* after 10 h of culture. On the left: Absorbance of viable cells. On the right: Micrographs of cells in culture.

Figure 7 shows that BLIS was not cytotoxic to peripheral blood mononuclear cells (PBMCs), the absorbance of viable cells treated with BLIS (0.9–1.0) being very close to that of untreated ones (control) (1.0–1.2). In contrast, it demonstrated cytotoxicity to human colon adenocarcinoma Caco-2cells, since the treatment led to a drop in the absorbance of viable cells from 2.7–3.0 in the control to only 2.1–2.3. Moreover, unlike PBMCs, the treatment with BLIS impaired the morphology of Caco-2 cells in culture**.**

## Cell death induction assay

**Figure 8:**
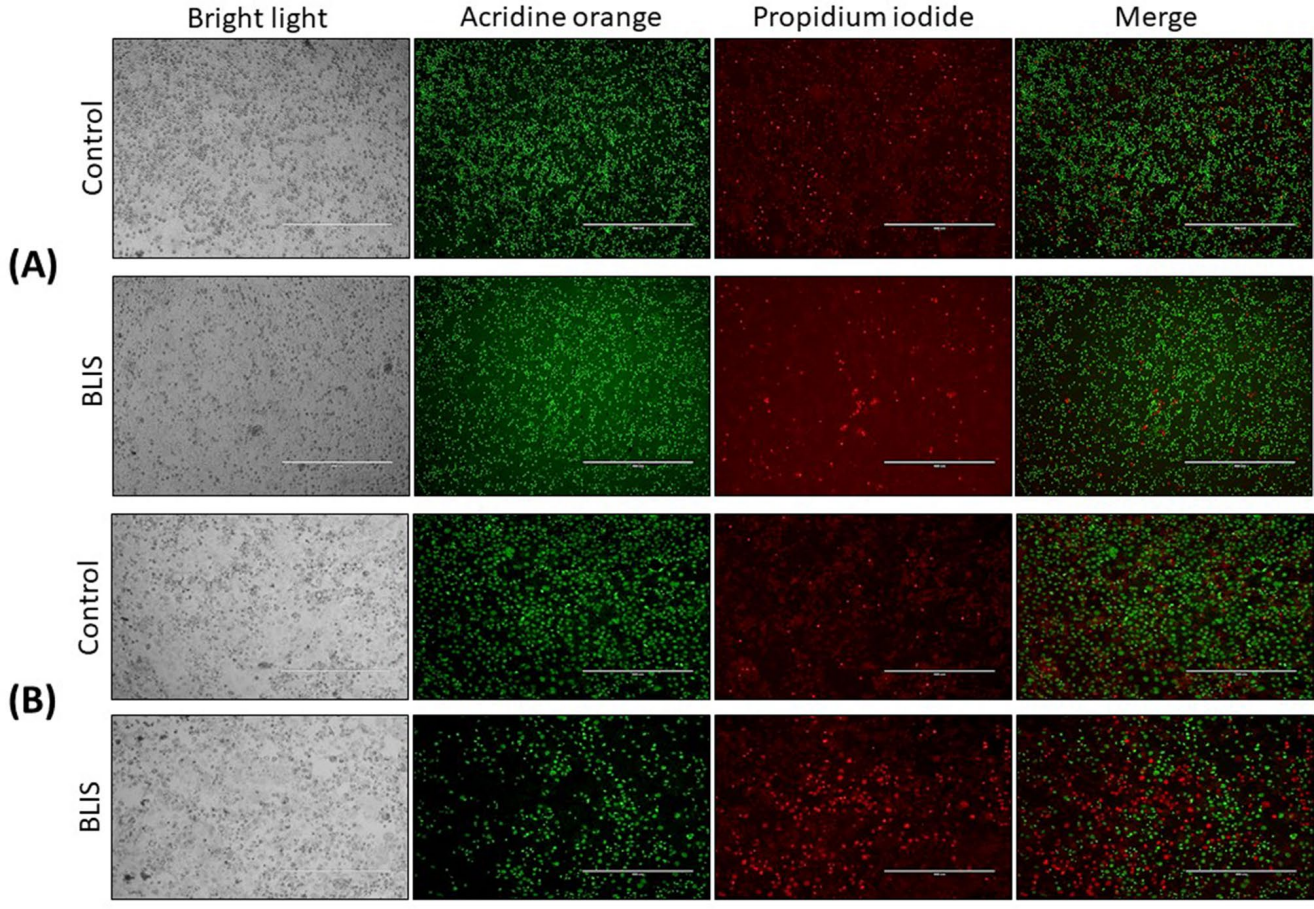
Induction of cell death in (**a**) peripheral blood mononuclear cells (PBMCs) and (**b**) human colon adenocarcinoma cells Caco-2 after 48 h of treatment with the BLIS produced by *P. pentosaceus* after 10 h of culture. Viable and dead cells are stained green and red, respectively.

It is evident in Fig. 8a that BLIS was not toxic to PBMCs, as the treated cells remained alive after 48 h of treatment with no relevant difference with the control. In contrast, as suggested by a significantly higher number of dead cells than in the control, BLIS was found to be toxic to Caco-2 cells after the same time (Fig. 8b). This result, which is quite encouraging since the targets are tumor cells, suggests possible applications of BLIS not only in food preservation, but also in medical-pharmaceutical applications.

## Conclusions

Novel antimicrobial molecules able to act as biopreservatives, instead of chemical preservatives, are of great interest for the feed and food industry mainly from the microbiological safety and food security viewpoints. *Pediococcus pentosaceus* LBM 18 BLIS inhibited the growth of spoilage bacteria belonging to *Lactobacillus*, *Enterococcus* and *Listeria* genera and effectively suppressed the growth of fungi isolated from corn grain silage for up to 25 days, impairing the morphology of their colonies likely affecting fungal membranes. The results of the present study showed the effectiveness of BLIS produced by the strain *P. pentosaceus* LBM 18 as an antibacterial and antifungal biomolecule, which may find possible applications in agriculture and food industry as a natural bio-controlling agent. Future efforts will deal with BLIS structural characterization and the mechanism of its interaction with target microorganisms.
